# Preclinical evaluation of the anti-tumor activity of pralatrexate in high-risk neuroblastoma cells

**DOI:** 10.18632/oncotarget.27697

**Published:** 2020-08-11

**Authors:** Rachael A. Clark, Sora Lee, Jingbo Qiao, Dai H. Chung

**Affiliations:** ^1^Department of Surgery, Division of Pediatric Surgery, University of Texas Southwestern Medical Center, Dallas, TX 75235, USA; ^*^These authors contributed equally to this work

**Keywords:** neuroblastoma, pralatrexate, N-myc, folate metabolism

## Abstract

Introduction: Pralatrexate is a folate analogue inhibitor of dihydrofolate reductase exhibiting high affinity for reduced folate carrier-1 with antineoplastic and immunosuppressive activities, similar to methotrexate. Despite advances in multi-modality treatment strategies, the survival rates for children with high-risk neuroblastoma have failed to improve. Therefore, the intense research continues in order to identify the ideal novel agent or combination of chemotherapy drugs to treat high-risk neuroblastoma.

Materials and Methods: Four human neuroblastoma cell lines were used to determine IC_50_ values of select chemotherapy agents. Antiproliferative effects of pralatrexate were assessed by adherent and non-adherent colony formation assays. Cell cycle arrest and apoptosis were measured by flow cytometry and immunoblotting. PDX tissue culture was used to assess *ex vivo* efficacy.

Results: Treatment with pralatrexate in all four neuroblastoma cell lines blocked cell growth in 2D and 3D culture conditions in a time-dependent manner. The potency of pralatrexate was ten-fold stronger than methotrexate, as measured by IC_50_. Pralatrexate-induced apoptosis was confirmed by caspase-3 activation and PARP cleavage. *MYCN* and *SLC19A1* mRNA expressions were decreased with pralatrexate in *MYCN*-amplified neuroblastoma cells.

Conclusions: Pralatrexate demonstrated effective inhibition of cell growth and viability. The higher potency of pralatrexate compared to methotrexate, a drug with high levels of toxicity, suggests pralatrexate may be a safer alternative to methotrexate as an effective chemotherapeutic agent in the treatment of patients with high-risk neuroblastoma.

## INTRODUCTION

Neuroblastoma is a pediatric tumor derived from neural crest cells. It is the most common pediatric solid tumor, accounting for approximately 15% of pediatric cancer deaths [1], and it typically presents as a painless abdominal mass in infants and toddlers of 18 to 22 months of age [2]. Poor prognostic factors in children with neuroblastoma include: age > 18 months at time of diagnosis, unfavorable histology, increased vascularization, and *MYCN* amplification [3]. Despite intense research focused on the biology of neuroblastoma, it remains one of the most enigmatic pediatric cancers in terms of its underlying molecular pathogenesis. There has been only incremental improvement in the overall survival of children with high-risk neuroblastoma, necessitating the search for a novel agent or combination of chemotherapy drugs [4].

Altered metabolism is key to cancer cell proliferation. Among the various metabolic pathways that are affected, folate metabolism plays an important role. Folate is essential for DNA synthesis and cell growth, especially in rapidly dividing cells. Inhibition of folate metabolism is the basis for many chemotherapy drugs. In neuroblastoma, folate mediated one-carbon metabolism is associated with aggressiveness and *MYCN* amplification [5]. A study by Lau et al. in 2015 demonstrated higher folate requirements in *MYCN* amplified neuroblastoma cells compared to non-*MYCN* amplified cells [6]. They also showed that the increased folate uptake is mediated by reduced folate carrier-1 (RFC-1), which is encoded by the gene *SLC19A1* [7]. Previous studies have demonstrated that *SLC19A1* is associated with *MYCN* amplification in neuroblastoma and that *SLC19A1* is a direct transcriptional target of N-myc [6]. The association between *MYCN* amplification and folate metabolism suggests the potential role of antifolate drugs in the treatment of neuroblastomas.

Methotrexate is a widely used inhibitor of folate metabolism. It inhibits dihydrofolate reductase (DHFR) and therefore disrupts purine and thymidylate biosynthesis, leading to inhibited DNA replication and cell death. However, in the 1970s methotrexate was found to have high levels of toxicity combined with low treatment response rates in neuroblastoma patients and therefore, it has not been clinically used for neuroblastoma treatment [6]. Pralatrexate is a folate analogue inhibitor of DHFR that exhibits high affinity for RFC-1 [8] and folylpolyglutamate synthetase (FPGS). Pralatrexate demonstrates antineoplastic and immunosuppressive properties that are similar to methotrexate. It was FDA approved in the United States for treatment of relapsed or refractory peripheral T-cell lymphoma in 2009 [9]. A study by Serova et al. in 2011 demonstrated decreased mRNA expression of *SLC19A1* and *SLC25A32*, a mitochondrial folate carrier, with pralatrexate treatment in several cancer cell lines [10]. As previously discussed, *SLC19A1* is down-stream target of N-myc in neuroblastoma. The high affinity of pralatrexate for the *SLC19A1* encoded RFC-1 protein may demonstrate a potential role in the treatment of *MYCN*-amplified neuroblastoma.

The development of a new chemotherapeutic regimen is a long process that can take years to enter clinical trials and subsequently into bedside therapy. Identifying alterative applications for previously FDA-approved drugs is a method that allows for quicker use in clinical practice [4]. Therefore, we sought to evaluate current FDA approved antineoplastic drugs as potential novel treatment strategies for high-risk neuroblastoma and set out to assess the inhibitory role of pralatrexate on neuroblastoma cells.

## RESULTS

### The IC_50_ of pralatrexate is ten-fold less than methotrexate

**Figure 1:**
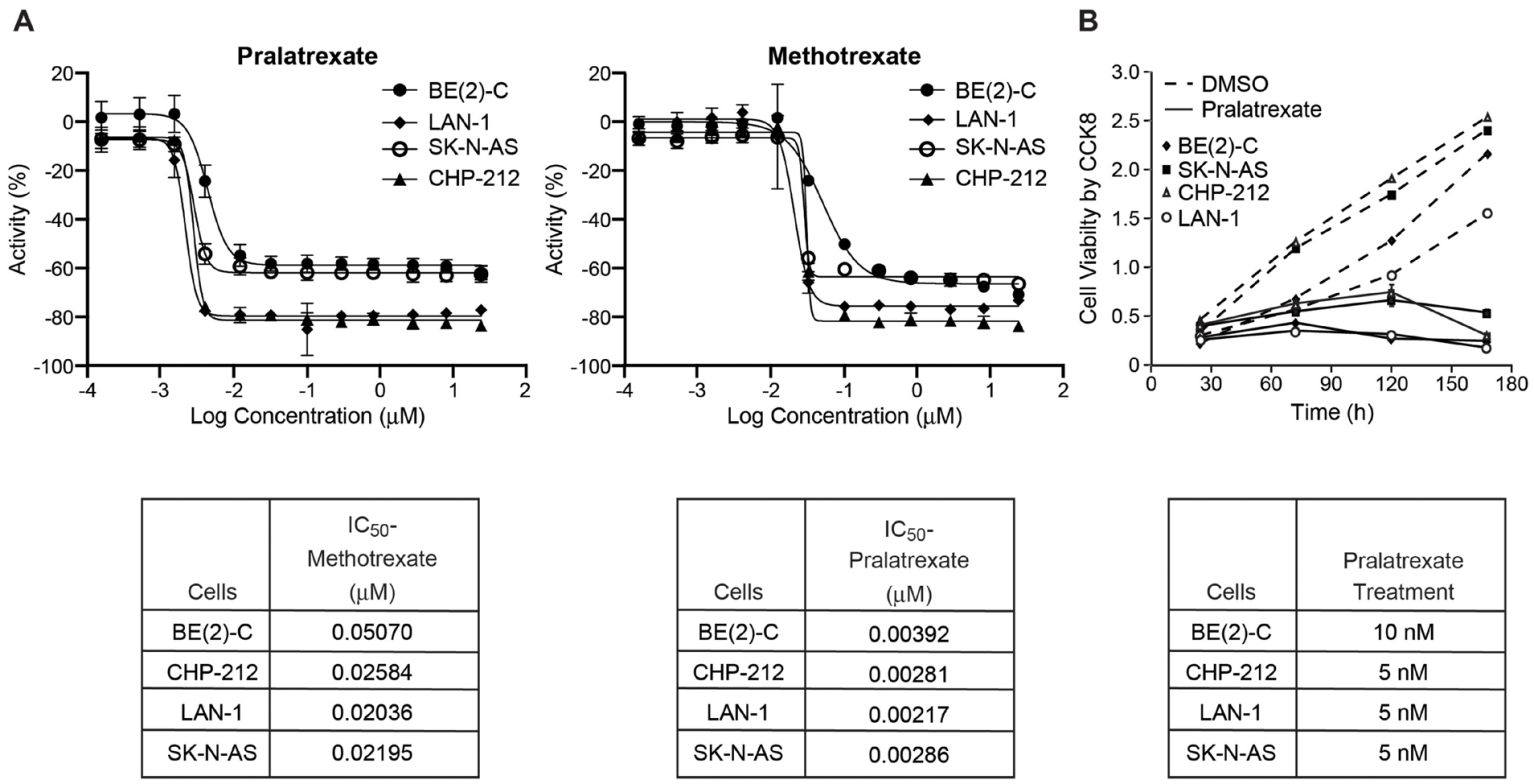
IC_50_ of human neuroblastoma cell lines BE(2)-C, CHP-212, and LAN-1 (*MYCN*-amplified) and SK-N-AS (non-*MYCN*-amplified). (**A**) The IC_50_ doses of BE(2)-C, CHP-212, LAN-1, and SK-N-AS cells treated with methotrexate were 0.05, 0.03, 0.02, and 0.02 μM respectively. The IC_50_ doses of BE(2)-C, CHP-212, LAN-1, and SK-N-AS cells treated with pralatrexate were 0.004, 0.003, 0.002, and 0.003 μM respectively. Cell growth was determined after 72 h of continuous exposure to methotrexate or pralatrexate (0.1 nM–25 μM), measured by Cell Titer Glo^™^ assay, and the IC_50_ of each drug was calculated for each cell line using the Genedata Screener software. Values shown are mean ± SD of three separate experiments. (**B**) Cell viability was assessed using the Cell Counting Kit-8 assay. Pralatrexate significantly inhibited cell viability in all four neuroblastoma cell lines as compared to DMSO control group.

Four human neuroblastoma cell lines including *MYCN*-amplified, BE(2)-C, CHP-212, and LAN-1, as well as the non-*MYCN* amplified cell line, SK-N-AS, were treated with methotrexate or pralatrexate. Cell growth was determined after 72 h of continuous exposure to methotrexate or pralatrexate (0.1 nM–25 μM), measured by Cell Titer Glo^™^ assay, and the IC_50_ of each drug was calculated for each cell line using the Genedata Screener software. Values shown are mean ± SD of three separate experiments. As shown in Figure 1A, in all four cell lines, the IC_50_ of pralatrexate was approximately ten-fold less than the IC_50_ of methotrexate. These data demonstrate that high-risk neuroblastoma cells have enhanced pralatrexate sensitivity compared to methotrexate and that pralatrexate inhibits both *MYCN* amplified and non-*MYCN* amplified neuroblastoma growth in the low nanomolar range *in vitro*.

### Pralatrexate inhibited neuroblastoma cell growth

**Figure 2:**
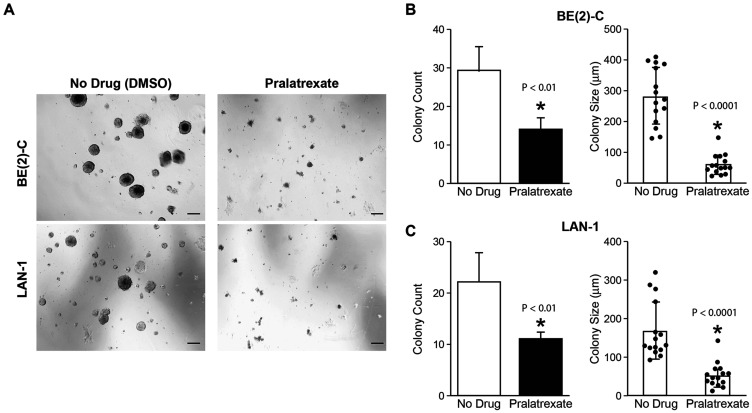
Pralatrexate inhibited neuroblastoma colony growth. (**A**) Representative images of light microscopy (4× magnification) for BE(2)-C and LAN-1 cells after 7 days of treatment with pralatrexate versus control. Pralatrexate treatment decreased cell growth in both BE(2)-C and LAN-1 cells compared to control (scale bar, 200 μm). (**B**) Colony count and colony size for BE(2)-C cells were analyzed and quantified (mean ± SD; ^*^=*p* < 0.05 for 10 nM pralatrexate treatment vs. no drug). (**C**) Colony count and colony size for LAN-1 cells were analyzed and quantified (mean ± SD; ^*^=*p* < 0.05 for 5 nM pralatrexate treatment vs. no drug).

To demonstrate the timing of growth inhibition, we treated neuroblastoma cells with pralatrexate (5 or 10 nM) and measured cellular viability over a time course of 4 days (Figure 1B). Significant cell growth inhibition was first noted by day 2 in SK-N-AS and CHP-212 cells, and by day 3 in LAN-1 and BE(2)-C cells. This indicates that pralatrexate effectively inhibits the proliferative potential of neuroblastoma cells. We further validated the effects of pralatrexate on neuroblastoma cells, *in vitro*, by quantifying colony growth in a 3D matrix hydrogel where cells grow and self-assemble into clusters. 3D cultures are more physiologically relevant and better represent *in vivo* tissue. BE(2)-C and LAN-1 cells are high colony-forming neuroblastoma cell lines [11]. Concurrent treatment with pralatrexate completely abolished the ability of BE(2)-C and LAN-1 cells to develop colonies in gel drops (Figure 2A). Both BE(2)-C and LAN-1 cell lines treated with pralatrexate demonstrated a decreased colony count and a decrease in colony size compared to cells treated with DMSO (Figure 2B and 2C). The colonies were counted from three separate microscopic fields and their size was measured using the scale bar on each image using Image J. These findings suggest that pralatrexate represses the tumorigenesis potential and tumor progression of neuroblastoma.

### Pralatrexate induced G1 phase cell cycle arrest, apoptosis, and decreased N-myc expression

**Figure 3:**
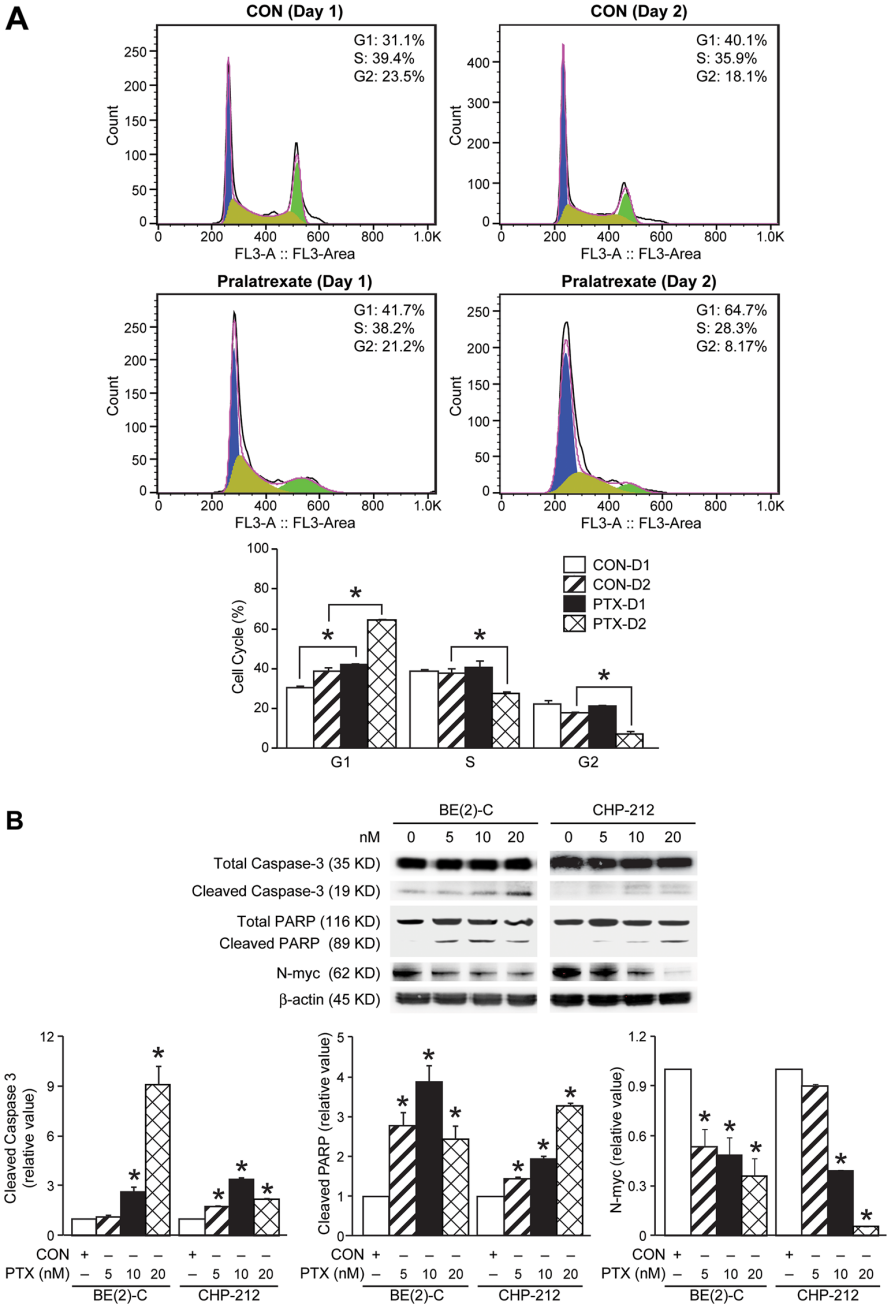
Effects of pralatrexate treatment on caspase-3, PARP, and N-myc protein expression. (**A**) Cell cycle analysis with propidium iodide demonstrates enhanced G1 cell cycle arrest at 24 and 48 h following treatment. Cell cycle analysis was completed with 10,000 events per replicate (mean ± SD; ^*^=*p* < 0.05 for 10 nM pralatrexate treatment vs. no drug). (**B**) Treatment with increasing doses of pralatrexate induced apoptosis in BE(2)-C and CHP-212 cells. Cells treated with pralatrexate demonstrated cleaved caspase-3 protein expression when treated with 10 and 20 nM doses. Cleaved PARP expression was noted after treatment with 5 nM. Treatment with pralatrexate decreased N-myc protein expression in BE(2)-C and CHP-212 cells. β-actin was used as an internal control.

To further test whether pralatrexate directly altered neuroblastoma cell proliferation, we evaluated the cell cycle distribution of treated cells compared with control. Cell cycle analysis was performed in BE(2)-C cells treated with pralatrexate (10 nM) or control at days 1 and 2 after treatment. We observed a significant, but modest, increase in the G1 phase of the cell cycle, ranging from a 10–24% increase in the G1 cell population, demonstrating induction of G1 cell cycle arrest (Figure 3A). Given the dramatic decrease in cell viability observed between days 2 and 3 of pralatrexate treatment (Figure 1B), we hypothesized that pralatrexate may also induce apoptosis in neuroblastoma cells. To confirm apoptosis in cells treated with pralatrexate, Western blotting was performed. BE(2)-C and CHP-212 cells were treated with increasing doses of pralatrexate (5, 10, and 20 nM). The protein expression of total and cleaved caspase-3, as well as total and cleaved PARP, were examined at each increasing dose of pralatrexate (Figure 3B), confirming the induction of apoptosis. Apoptosis was also seen secondary to pralatrexate treatment in non-*MYCN* amplified cells, SK-N-AS, SK-N-SH, and SH-SY5Y (Supplementary Figure 1). Given the proliferating role of N-myc in neuroblastoma tumorigenesis, we next evaluated whether pralatrexate could alter the N-myc expression. Interestingly, we found decreased N-myc expression with increasing doses of pralatrexate in both BE(2)-C and CHP-212 cells (Figure 3B), demonstrating persistent defects in proliferative potential induced by pralatrexate in neuroblastoma.

### Pralatrexate decreased *MYCN* and *SLC19A1* gene expressions compared to *SLC25A32*

**Figure 4:**
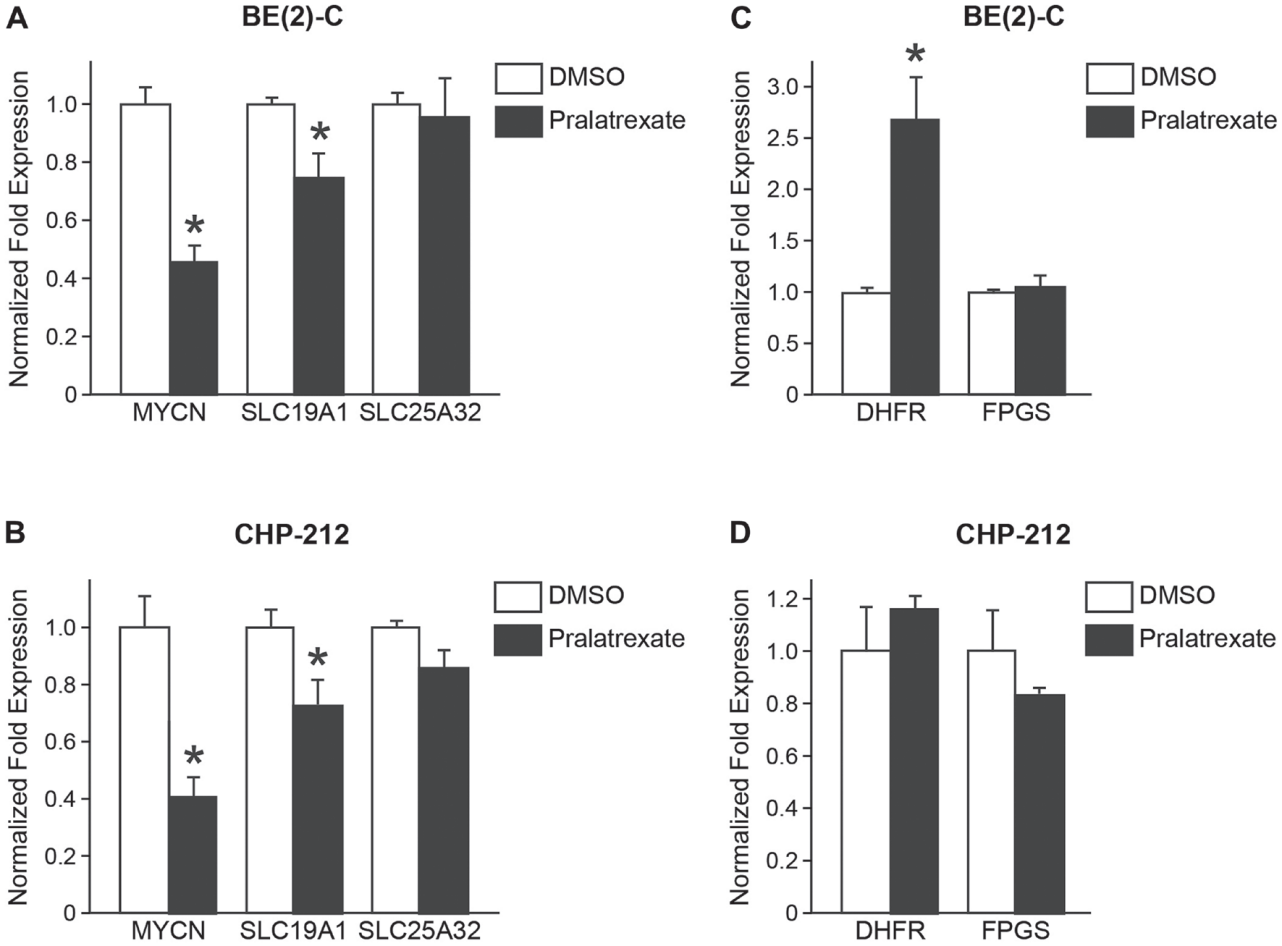
Effects of pralatrexate treatment on *MYCN*, *SLC19A1*, and *SLC25A32* gene expressions. After 1 day of treatment, the mRNA expression of *MYCN, SLC19A1*, and *SLC25A32* were measured by qPCR in (**A**) BE(2)-C cells treated with 10 nM of pralatrexate when compared with DMSO treated cells, and in (**B**) CHP-212 cells treated with 5 nM of pralatrexate. (**C**, **D**) qPCR was performed on BE(2)-C and CHP-212 cells treated with pralatrexate to assess for mRNA expression of two key-enzymes in folate synthesies, *DHFR* and *FPGS* in the treatment (mean ± SD; ^*^=*p* < 0.05 for pralatrexate treatment vs. no drug).

Previous studies demonstrated that the expression of *SLC19A1*, the gene encoding the RFC-1 receptor, is associated with *MYCN* amplification in neuroblastoma [6]. Pralatrexate is a folate analogue inhibitor with high affinity for RFC-1 [8]. Therefore, we sought to examine the effects of pralatrexate treatment on *MYCN* and *SLC19A1* gene expression in BE(2)-C and CHP-212 cell lines compared to the effects in non-*MYCN* amplified cells. BE(2)-C and CHP-212 cells were treated with 10 and 5 nM pralatrexate or DMSO and qPCR was performed. As expected from the finding in Figure 3B, pralatrexate treatment resulted in decreased *MYCN* expression in both BE(2)-C and CHP-212 cells (Figure 4A and 4B). Interestingly, we also found that both BE(2)-C and CHP-212 cells treated with pralatrexate demonstrated decreased *SLC19A1* expression, but no difference in *SLC25A32* expression, compared to control cells (Figure 4A and 4B). Treatment of non-*MYCN* amplified cells, SK-N-AS, SK-N-SH, and SH-SY5Y, did not affect *SLC19A1* or *SLC25A32* expression (Supplementary Figure 2). These findings may support the previous studies [6, 10] that *SLC19A1* is a direct transcription target of *MYCN* in neuroblastomas, and pralatrexate treatment affects *SLC19A1* expression in *MYCN* amplified cells, but not *SLC25A32* expression. In addition, in both BE(2)-C and CHP-212 cells, pralatrexate did not decrease *FPGS* mRNA expression (Figure 4C and 4D). Neither *SLC25A32* or *FPGS* expression was affected in non-*MYCN* amplified cells treated with pralatrexate (Supplementary Figure 2). Interestingly, in the BE(2)-C cells and the non-*MYCN* amplified cells, pralatrexate treatment led to an increase in *DHFR* expression. This may imply treatment with pralatrexate selected for cells with increased DHFR expression and inherent pralatrexate resistance or a resultant upregulation of the *DHFR* gene with DHFR protein inhibition. These findings would be consistent with findings by Serova et al. in which pralatrexate-resistant cells demonstrated increased DHFR protein expression [10].

### Pralatrexate treatment response in *ex vivo* neuroblastoma growth

**Figure 5:**
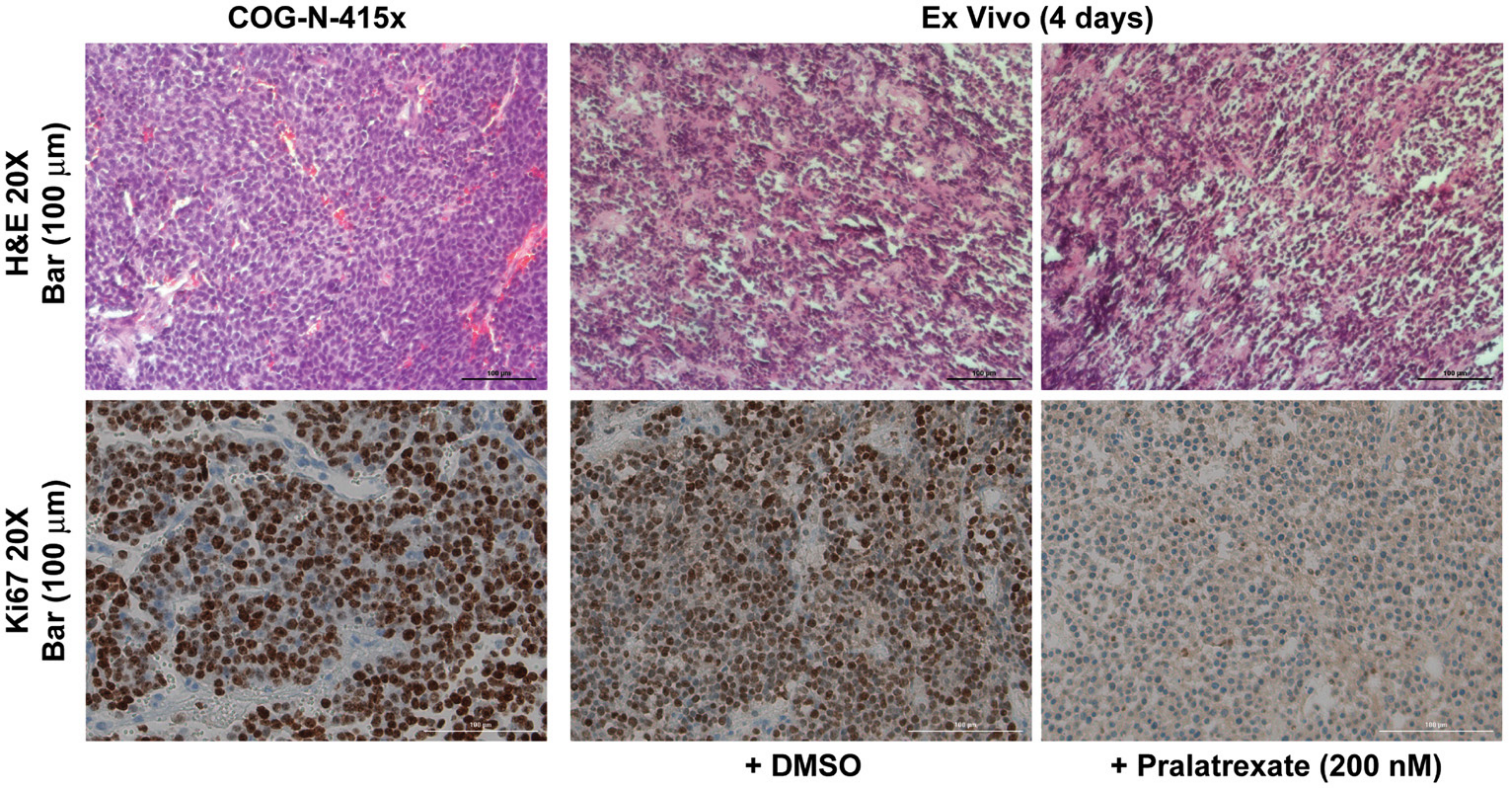
*Ex vivo* tissue culture model recapitulated antitumor response to pralatrexate. Representative H&E and Ki67 immunohistochemistry staining sections were obtained from a neuroblastoma PDX, COG-N-415× treated *ex vivo* with 200 nM pralatrexate or vehicle control for 4 days, and demonstrated poorly differentiated neuroblastoma cells and decreased Ki67 staining in pralatrexate-treated tumor compared to vehicle control (20× magnification, scale bar, 100 μm).

Given these persistent *in vitro* findings, we next evaluated whether pralatrexate could be evaluated *ex vivo* to guide treatment decisions for individual patients. We used a neuroblastoma PDX model where tumors were dissected into 1-mm^3^ pieces and cultured in duplicate on a presoaked gelatin sponge in 24-well plates containing 500 μL RPMI 1640 with 10% FBS, antibiotic/antimycotic solution, 0.01 mg/mL hydrocortisone, and 0.01 mg/mL insulin. The size of these fragments is comparable to the size of a standard clinical tumor biopsy specimen. The fragments were then cultured for 4 days in the presence of pralatrexate (200 nM) using standard cell culture conditions. Notably, tumor tissues were significantly affected by *ex vivo* pralatrexate treatment and showed decreased Ki67 staining compared to tissues cultured in vehicle control treatment (Figure 5). These results suggest that a simple short-term *ex vivo* treatment assay of a viable tumor specimen may aid in identifying neuroblastoma patients who are likely to gain benefit from pralatrexate treatment options in the future.

## DISCUSSION

High-risk neuroblastoma remains quite difficult to cure, necessitating the discovery of new chemotherapy agents to be used alone or in combination therapy. Previous studies have reported an increased folate demand in *MYCN*-amplified neuroblastoma cells mediated by the RFC-1 receptor [6]. Additionally, the gene encoding the RFC-1 receptor, *SLC19A1*, is a direct transcriptional target of N-myc in neuroblastoma cells, suggesting a role for anti-folate drugs in the treatment of neuroblastoma. Methotrexate has previously been studied in neuroblastoma, however, it was found to have a prohibitive toxicity and has not been used in neuroblastoma clinically. In contrast, pralatrexate, a folate analogue inhibitor, is similar to methotrexate with a more favorable side effect profile suggesting a potential role for the use of pralatrexate as a chemotherapeutic agent against neuroblastoma.

The present study sought to determine the effects of treatment with pralatrexate on *in vitro* and *ex vivo* cell growth in four human neuroblastoma cell lines. The IC_50_ of pralatrexate was found to be 10-fold less than that of methotrexate. This ten-fold difference between pralatrexate and methotrexate was also found in colon, breast, and thyroid cancer cells, by Serova et al. in 2011 [10]. The decreased IC_50_ of pralatrexate allows for treatment with lower doses and a more tolerable side-effect profile compared to methotrexate, independent of *MYCN* amplification.

Pralatrexate not only induced cell-death via apoptosis, but it also successfully inhibited neuroblastoma *in vitro* cell growth and proliferation in 2D and 3D cell cultures as well as in our PDX *ex-vivo* model. By inhibiting the RFC-1 receptor, pralatrexate decreased the amount of folate entering cells and in turn, decreased DNA synthesis. This was demonstrated by the increased time spent in the G1-phase of the cell cycle in cells treated with pralatrexate. PDXs have been shown to parallel clinical outcome in various tumor types [12]. The major applications of neuroblastoma PDXs would be related to drug testing, exploration of treatment resistance, and biomarker discovery. Combining PDXs and *ex vivo* culture will incorporate human tumor tissue in its native 3D state and enable dynamic manipulation of the system minimizing animal experiments and cost.

Lau et al. has shown that *SLC19A1* is a down-stream direct transcriptional target of N-myc in neuroblastoma cells and that *MYCN*-amplified cells have an increased folate dependence [6]. In our study, pralatrexate also led to a decrease in expression of the RFC-1 genes, *SLC19A1* and *SLC25A32*. The decrease in *SLC19A1* was more pronounced compared to the mitochondrial folate receptor gene, *SC25A32*, suggesting pralatrexate may be more specific to the cytosolic RFC-1 receptor compared to the mitochondrial RFC-1 receptor. However, further studies are necessary to investigate this relationship. Meanwhile, pralatrexate treatment led to a marked increase in *DHFR* expression in BE(2)-C cells and a slight increase in CHP-212 cells. This is unlikely an upregulation of *DHFR* and more indicative of increased *DHFR* mRNA being harvested from pralatrexate resistance cells. Similar results were found in a previous study on colon, breast, and thyroid cancer cells lines. Serova et al. found that pralatrexate-resistant cells had increased DHFR protein expression [10]. The increase in *DHFR* expression may lead to an increase in the amount of DHFR protein requiring more than 10 nM of pralatrexate to inhibit cell growth and proliferation. However, further studies surrounding the dose of pralatrexate and its relationship to *DHFR* gene expression are needed.

Neuroblastoma is a heterogenous tumor and further studies are needed to examine the effects of pralatrexate on additional cells lines. Additionally, the remaining cells that survived after pralatrexate treatment may represent pralatrexate resistant cells. Future studies are needed to elucidate potential mechanisms of pralatrexate resistance such as increased *DHFR* gene expression, as well as the relationship between pralatrexate and *SLC19A1* versus other folate synthesis enzyme expressions. Given pralatrexate is already an FDA-approved and in clinical use, future clinical studies are needed to investigate the effects of pralatrexate treatment on neuroblastoma *in vivo*.

## MATERIALS AND METHODS

### Cells, antibodies and reagents

The neuroblastoma cell line, LAN-1, was a gift from Dr. Robert C. Seeger (University of Southern California, Los Angeles, CA). All other neuroblastoma cell lines BE(2)-C, CHP-212, SK-N-AS, SK-N-SH, and SH-SY5Y were purchased from the American Type Culture Collection (ATCC, Manassas, VA). Cells were maintained in RPMI 1640 with glutamine and 10% FBS at 37°C in a humidified atmosphere consisting of 5% CO_2_ and 95% air. Primary antibodies for Caspase-3 (1:1000, Cat No 9662), PARP (1:1000, Cat No 9542), N-myc (1:500, Cat No 9405), were purchased from Cell Signaling Technology (Danvers, MA), Ki-67 (1:200, Cat No 16667) was from Abcam (Cambridge, MA), and β-actin (1:1000, Cat No A2066) was from Sigma-Aldrich (St. Louis, MO). Methotrexate and pralatrexate were obtained from National Cancer Institute/Division of Cancer Treatment and Diagnosis/Developmental Therapeutics Program: http://dtp.cancer.gov, and dissolved in dimethyl sulfoxide (DMSO), and further diluted in culture media to desired concentrations. Neuroblastoma COG-N-415× patient-derived xenograft (PDX) cells were obtained from the Childhood Cancer Repository maintained by the Children’s Oncology Group (COG) and Xenograft Repository.

### Drug sensitivity and dose responsive curve assay

For cell viability screening, cells [BE(2)-C:1500/well, LAN-1: 3000/well; CHP-212:4000/well; SK-N-AS: 3000/well] were plated and treated the following day with methotrexate and pralatrexate. Cell growth was determined after 72 h of continuous exposure to 0.1 nM–25 μM of methotrexate or pralatrexate using Cell Titer Glo^TM^ reagent (Promega), with luminescence measured using an EnVision multi-label plate reader (Perkin-Elmer, Inc.).

### Cell viability assay

Neuroblastoma cells were seeded onto 96-well plates, permitted to attach overnight and were treated with either pralatrexate (5 or 10 nM) or DMSO for 4 days. Cell viability measurements using the Cell Counting Kit-8 (Dojindo Molecular Technologies, Inc, Rockville, MD) were obtained daily.

### 3D colony formation assay

BE(2)-C or LAN-1 cells were trypsinized, embedded in 35 μl of Cultrex^®^ RGF BME Type 2 matrix hydrogel (Trevigen, Gaithersburg, MD), and seeded in 48-well plates (200 cells/well). RPMI 1640 medium containing 10% FBS was added with pralatrexate treatment and incubated for 7 days. Colonies were photographed and the number and size were quantified. The colonies were counted from three separate microscopic fields and their size was measured by the scale bar on each image using Image J.

### Cell cycle analysis

Cell cycle distribution was analyzed using flow cytometry with propidium iodide (Sigma Aldrich). BE(2)-C cells were plated at equal numbers (1 × 10^6^ cells) and treated with either pralatrexate (10 nM) or DMSO. At day 1 and 2 after treatment, cells were washed and fixed in 70% ethanol. Fixed cells were incubated with 100 mg/mL RNAase for 30 minutes at 37°C, stained with propidium iodide (50 mg/ mL), and analyzed on a BD FACSCalibur (BD Biosciences, San Jose, CA).

### qPCR and immunoblotting

Total RNA was isolated and purified using a *TRIzol*^®^ Reagent (Thermo Scientific). cDNA was synthesized using the qScript cDNA SuperMix (QuantaBio). Real-time PCR and data collection were performed on a CFX96 instrument (Bio-Rad). Data were normalized to an endogenous control, β-actin. Specific target primers are: *MYCN* (forward 5ʹ-GCTTCTACCCGGACGAAGATG-3ʹ; reverse 5ʹ-CAG CTCGTTCTCAAGCAGCAT-3ʹ), *SLC19A1* (forward 5ʹ-AACAGGTCTGGGTTTTGTGC-3ʹ; reverse 5ʹ-GTGCAGTATCATGCCCTGTG-3ʹ), *SLC25A32* (forward 5ʹ-ATTGGTGGAAGCTGATTTGC-3ʹ; reverse 5ʹ-TGGTCTGGATTTGGTCAACA-3ʹ), *DHFR* (forward 5ʹ-CTCAAGGAACCTCCACAAGG-3ʹ; reverse 5ʹ-GTTTAAGATGGCCTGGGTGA-3ʹ), *FPGS* (forward 5ʹ-GGGTGACCCTCAGACACAGT-3ʹ; reverse 5ʹ-GTCTTCAGGCCATAGCTTCG-3ʹ). Amplification was performed for 40 cycles of 30 s at 95°C, 30 s at 58°C, and 40 s at 72°C. Whole cell lysates were collected using cell lysis buffer and equal amounts of protein were loaded on a NuPAGE 4–12% Bis-Tris gel, followed by transfer onto PVDF membranes (Bio-Rad, Hercules, CA, USA), and probed with antibodies.

### *Ex vivo* culture and immunohistochemistry

COG-N-415× patient-derived xenograft cells were obtained from the Childhood Cancer Repository maintained by COG. Clinical and genomic features of the tumors were detailed in a study by Harenza et al. in 2017 [13]. Cells were suspended in Matrigel diluted 1:2 with PBS and 5 × 10^5^ cells were injected into the flank of NOD *scid* gamma mice at 5–6 weeks of age (UTSW Mouse Breeding Core). All studies were approved by the Institutional Animal Care and Use Committee at University of Texas Southwestern Medical Center. To keep cost down we used only female mice for passing PDX. Mice were euthanized once tumors reached 15 mm and tumors were dissected into 1-mm^3^ pieces and cultured in duplicate on a presoaked gelatin sponge (Johnson and Johnson) in 24-well plates containing 500 μL RPMI 1640 with 10% FBS, antibiotic/antimycotic solution, 0.01 mg/mL hydrocortisone and 0.01 mg/mL insulin (Sigma-Aldrich). Tissues were cultured at 37°C for 4 days with either pralatrexate (200 nM) or vehicle control, then formalin-fixed and paraffin embedded. COG-N-415× tissue sections were stained with hematoxylin and eosin or with an antibody against Ki-67.

### Statistical analysis

All results are shown as the mean value ± SD; statistical analyses were performed using student *t*-test for comparisons between the groups. A *p* value of < 0.05 was considered significant. GraphPad’s Prism 8.0 software was used for the statistical analysis.

## SUPPLEMENTARY MATERIALS



## Abbreviations

COG: Children’s Oncology Group
DHFR: dihydrofolate reductase
DMSO: dimethyl sulfoxide
FPGS: folylpolyglutamate synthetase
PDX: patient-derived xenograft
RFC-1: reduced folate carrier-1
