# Supplementary material for: Preclinical evaluation of the anti-tumor activity of pralatrexate in high-risk neuroblastoma cells
Source: Oncotarget. 2020 Aug 11;11(32):3069–77. doi: 10.18632/oncotarget.27697 (PMC7429182; doi:10.18632/oncotarget.27697)
Supplement: Supplementary file 1 [file oncotarget-11-3069-s001.pdf]

Preclinical evaluation of the anti-tumor activity of pralatrexate in high-risk neuroblastoma cells

SUPPLEMENTARY MATERIALS

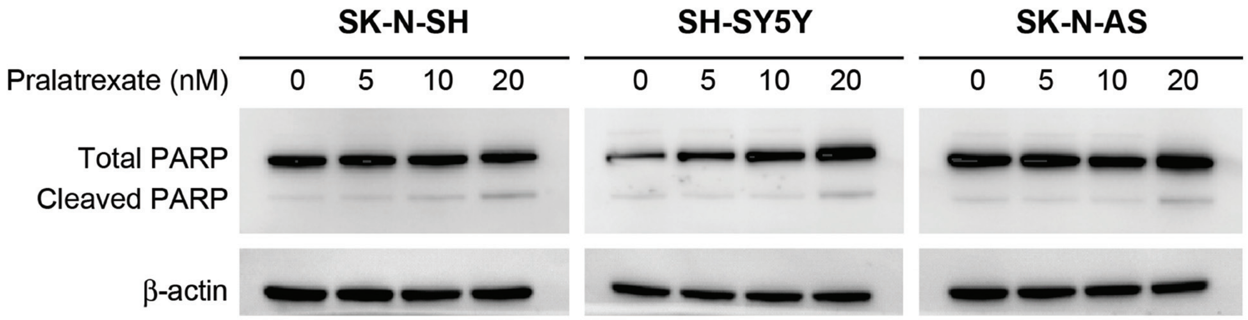

Supplementary Figure 1: Treatment with increasing doses of pralatrexate induced apoptosis in SK-N-SH, SH-SY5Y, and SK-N-AS cells. Cells treated with pralatrexate demonstrated cleaved PARP protein expression when treated with 20 nM dose after 24 h when compared with DMSO treated cells. β-actin was used as an internal control.

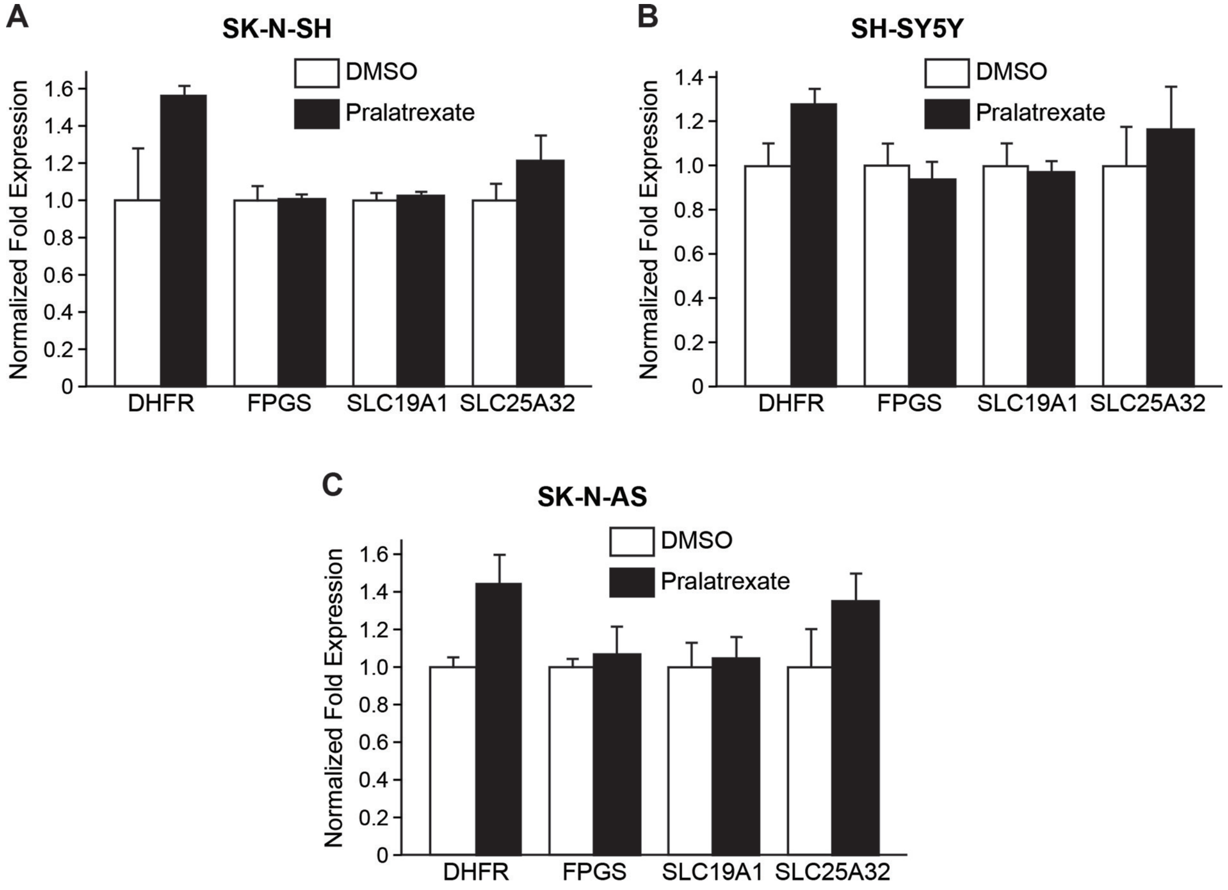

Supplementary Figure 2: qPCR survey for the expression of key carriers and enzymes in folate synthesis in SK-N-SH (**A**), SH-SY5Y (**B**) and SK-N-AS (**C**) cells. *SLC19A1*, *SLC25A32*, *DHFR* and *FPGS* were analyzed in 5 nM of pralatrexate treatment after 24 h when compared with DMSO treated cells. β-actin expression was used for the normalization.
